# Arthroscopic assistance during open reduction and fixation for complex acetabular fractures

**DOI:** 10.1007/s00590-023-03663-2

**Published:** 2023-08-08

**Authors:** Alessandro Aprato, Matteo Giachino, Alessandra Cipolla, Alessandro Massè

**Affiliations:** 1 Department of Surgical Sciences, University of Turin, Piazza Polonia, 94, 10126 Turin, TO Italy; 2 Department of Orthopedics, Turin Trauma Center (CTO), Turin, Italy

**Keywords:** Acetabular fracture, Hip arthroscopy, Arthroscopic-assisted reduction

## Abstract

Achieving an anatomical reduction in acetabular fracture is essential but may also be challenging. Most of complex fractures are treated with anterior approaches without direct visualization of the acetabular surface. In this paper, we present the surgical technique for arthroscopic assistance during open reduction and fixation for complex acetabular fractures. To our knowledge, this technique has not been described in the literature yet.

## Introduction

Anatomic reduction of acetabular fractures is challenging but essential, as several studies have shown that the quality of the reduction is the most important predictive factor influencing clinical outcome [1–3]. Matta et al. [4] demonstrated that a poor fracture reduction was the most negative prognostic indicator for clinical outcomes and argued that surgeons should strive for anatomic reductions and a congruent acetabular roof.

Anterior intrapelvic approaches (AIP), such as the ilioinguinal or modified Stoppa, are frequently used to reduce and fix complex acetabular fractures; however, these approaches do not allow direct visualization of the acetabular surface, leaving surgeons to rely on intraoperative fluoroscopy to judge reduction of the acetabulum [5, 6].

Recently, arthroscopic-assisted reduction and fixation of intraarticular fractures has been proposed as a way to improve reductions and outcomes across multiple types of fractures, such as posterior wall fractures [7–9] or pipkin type I femoral head fractures [10].

Arthroscopic-assisted reduction of complex acetabular fractures treated through an AIP approach has not yet been described, however. The purpose of this manuscript was to present such a technique for treating acetabular fractures with impacted articular fragments.

## Surgical technique

**Fig. 1:**
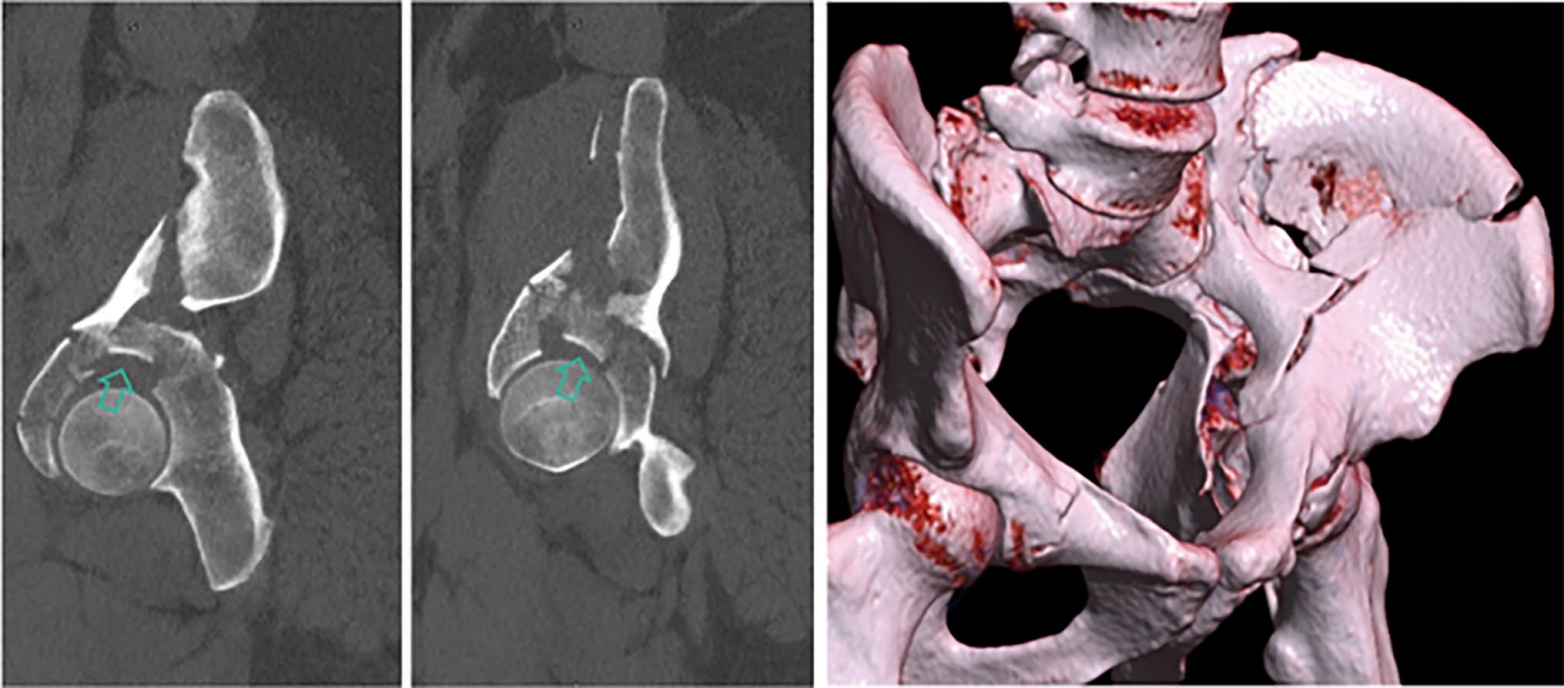
A 28-year-old man with severe obesity (BMI 367 kg/m^2^) fall from two meters’ height at work with direct trauma to the left side. The X-ray and the CT scan show a both column acetabular fracture with a large osteochondral fragment of the displaced acetabular roof (Fig. 1). Clinically there was a sciatic nerve injury associated. The large osteochondral fragment of the displaced acetabular roof is shown

The indication for an arthroscopic-assisted reduction of an acetabular fracture being treated through an AIP approach in our practice was the presence of an impacted articular fragment in the weight-bearing portion of the acetabular roof (Fig. 1). Preoperative treatment is performed according to the national guidelines [11]. A computed tomography (CT) scan and three-dimensional (3D) reconstruction are obtained for preoperative planning [2]. The choice of surgical approach is dictated by the need to visualize and reduce displaced fractured column(s) [12]: Ilioinguinal approach is a standard extrapelvic approach described by Letournel [13] which allows for a visualization of the anterior wall and column fractures; the modified Stoppa approach described by Cole et al.[14] allows for visualization of the inferior parts of the anterior column, the medial pelvic brim, and the quadrilateral surface, which can also be combined with the first window of an ilioinguinal approach. No changes in these approaches have been made since arthroscopic assistance has been introduced.

### Patient position and setup

**Fig. 2:**
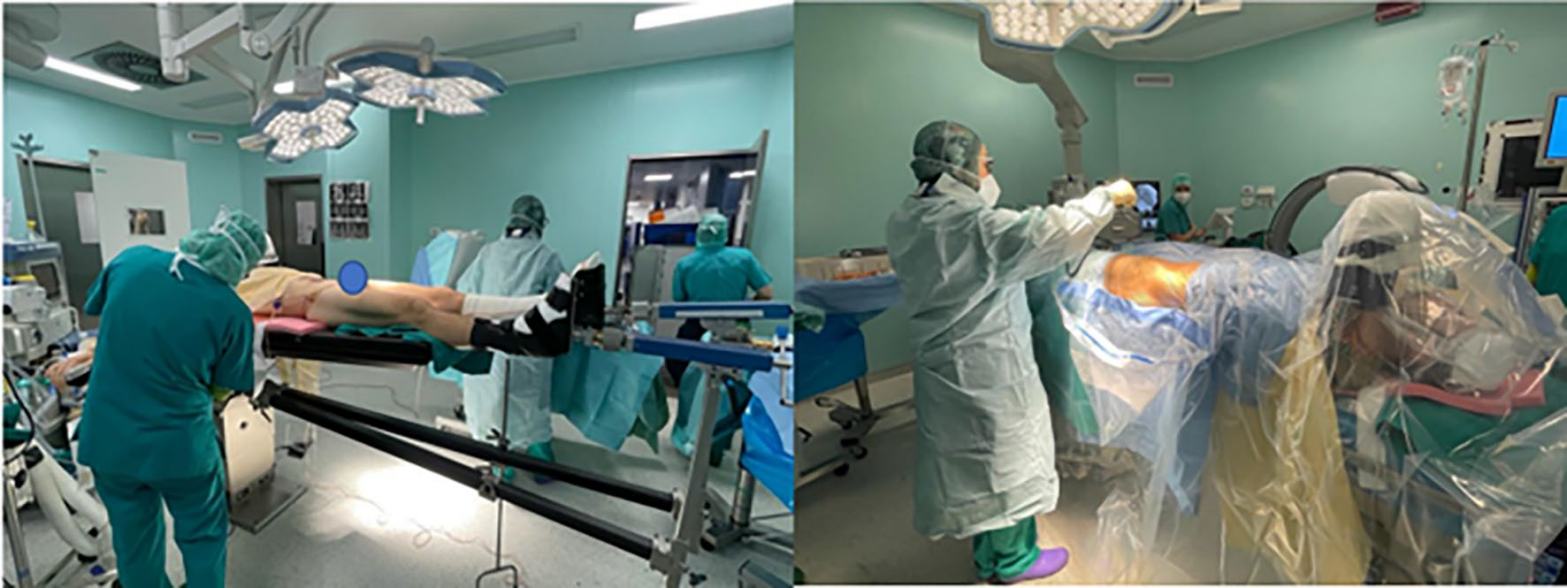
Patient position and setup

The patient is placed in supine position under general anesthesia on a radiolucent traction bed. A postless traction technique with dedicated surgical pads is utilized [15]. The arthroscopy video monitor is placed on the contralateral side, proximal to the patient’s head. The C-arm fluoroscopy machine is also positioned on the contralateral side, distal to the arthroscopy monitor (Fig. 2). Standard arthroscopy instruments are required, including a 17-gauge 6-inch spinal needle, trocar, nitinol guide wire, hip cannula, obturator, arthroscopy pump, arthroscope, radiofrequency ablator, and tissue shaver.

For the open approach, we utilize a cannulated ram, 1.6 K wire, Matta Pelvic System set, ball-spike pushers, reduction forceps, Jungbluth clamps, 6.5-mm cannulated screws, and allograft bone [16, 17].

### Surgical approach

A complete or partial ilioinguinal approach [13] is performed using the symphysis pubis and the anterior superior iliac spine landmarks for the incision. According to fracture pattern, surgeons decide on which of the three windows to develop. The first or lateral window extends lateral to the iliopsoas muscle, providing access to the quadrilateral plate, sacroiliac joint, and iliac wing. The second or middle window is developed between external iliac vessels and the iliopsoas offering access to the pelvic brim, quadrilateral plate, and a portion of the superior pubic ramus [18]. A fourth window (the modified Stoppa approach described by Cole et al. [14]) may also be used.

**Fig. 3:**
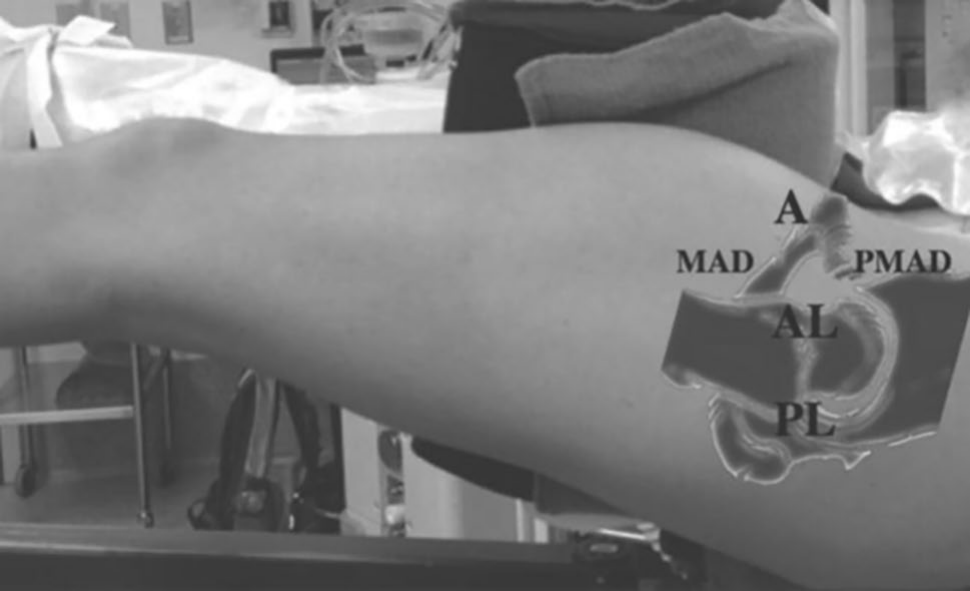
Principal arthroscopic portals [19]

The fracture is first exposed through the AIP. Traction is then applied, and the arthroscopic portals are made. The anterolateral portal is performed first with the standard technique using needle and nitinol wire , and then the arthroscope is introduced [19]. Under direct visualization, an anterior portal is added. If necessary, a third posterior portal can be made to help with hematoma removal (Fig. 3).

A fluid pump is used without exceeding 50 mmHg of pressure to avoid fluid extravasation until the fracture hematoma has been removed from the hip joint [20]; after hematoma removal, the fluid is aspirated and dry arthroscopy is utilized during reduction.

Arthroscopic examination of the central compartment of the hip joint is performed, and osteochondral fragment of the displaced acetabular roof is visualized (Fig. 1). A 360° evaluation is performed to evaluate for additional injuries, i.e. intraarticular lesions such as labral tears. If a labral tear is identified, it may be sutured at the end of the procedure. Small intraarticular fragments can also be removed prior to fracture reduction.

### Fracture reduction

**Fig. 4:**
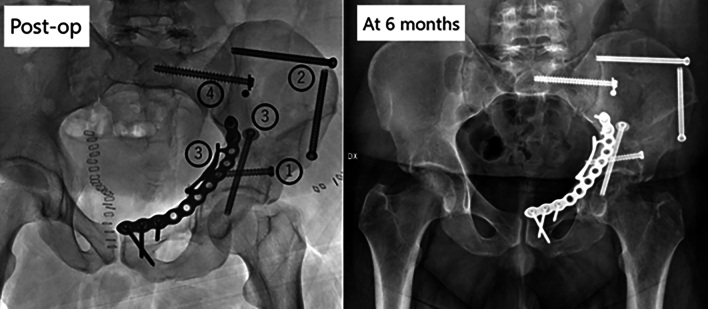
Immediate postoperative X-ray and follow-up at 6 months

Once the impacted fragment is identified under arthroscopy, its edges are exposed and the fragment mobilized. A supracetabular 1.6-mm K-wire is then placed under fluoroscopic guidance through the AIP approach, aimed at the impacted fragment. A cannulated ram is placed over the K wire, and the depressed fragment is reduced under arthroscopic visualization (Fig. 4). A slight overcorrection of the fragment, as described for tibial plateau impacted fragment reduction, is typically obtained [21].

### Fracture fixation

**Fig. 5:**
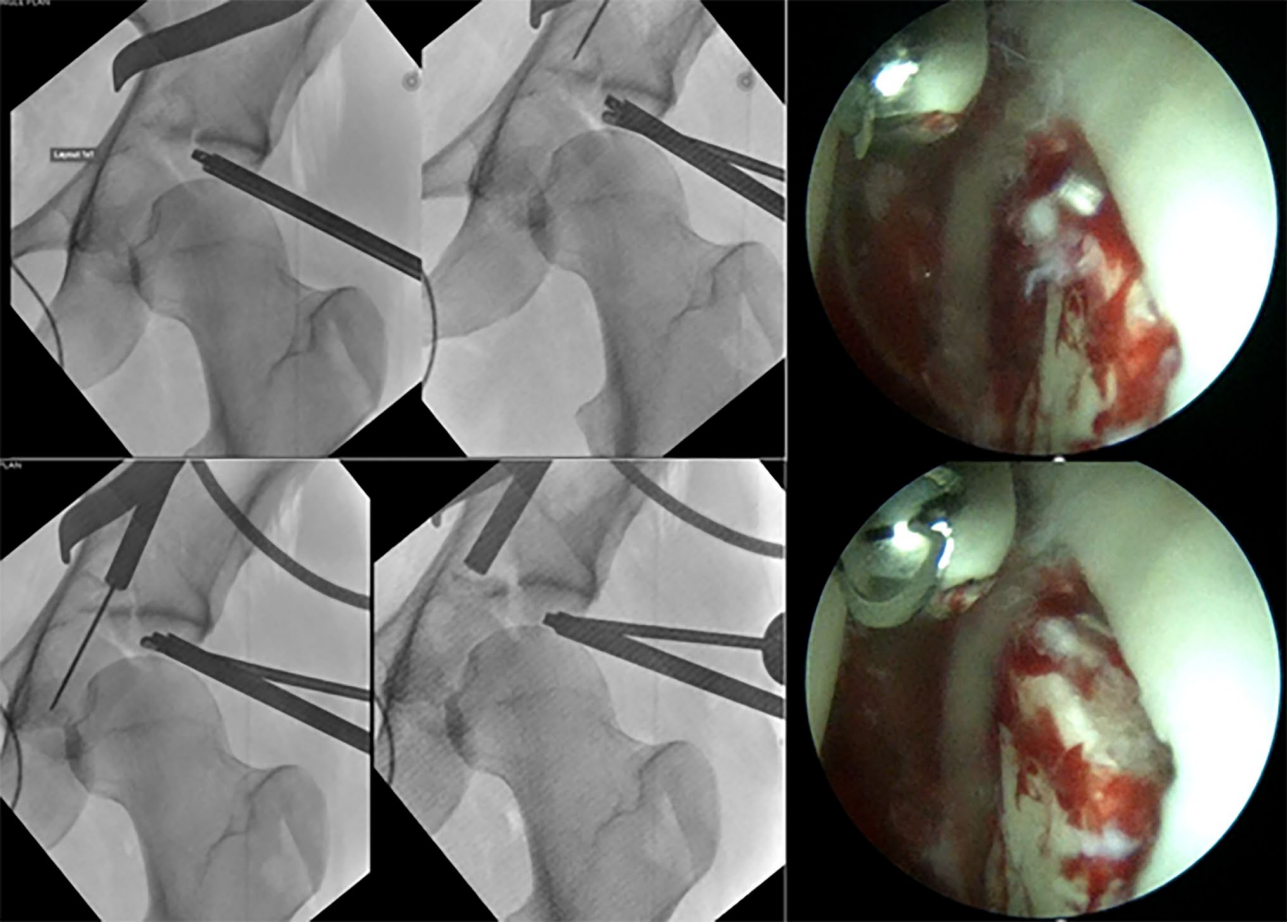
A healthy 21-year-old man presented to our ER after a crush trauma at work under a 500 kilos weight. The X-Ray and CT scan show an anterior column fracture plus posterior hemitransverse compound fracture (ACPHT) with joint depression of the acetabular roof. An AIP approach and hip arthroscopic assistance were performed. The patient was set up in supine position under general anesthesia on a carbon orthopedic traction bed. The fracture of the anterior column was identified through the AIP approach. The medial roof fracture and an important depression of the articular surface were identified through arthroscopic assistance. Supracetabular K-wire was placed under fluoroscopic control and proximal cortical flap was prepared to aid the fragment reduction. A cannulated ram was placed over the K wire and the depressed fragment was pushed stepwise until an anatomic articular reduction is achieved. At the end hypercorrection of the fragment was obtained as planned and synthetic bone was placed to fill the depression. The movement is continuously supervised by the arthroscope. Furthermore, the reduction of the fracture is evaluated by fluoroscopy. Surgery was performed in two hours and 30 min

After the achievement of fragment reduction, its position is supported by filling the acetabular defect with allograft cancellous bone cubes and secured in place with a percutaneous 6.5 mm cannulated “Magic” screw [22]. Reduction and fixation of the remaining fracture patterns is then performed utilizing standard non-arthroscopic assisted techniques (Fig. 5).

### Postoperative care

Arthroscopic-assisted reductions to not alter our standard postoperative protocols. Patients are typically made non-weight bearing on the affected extremity for 12-weeks.

## Discussion

**Fig. 6:**
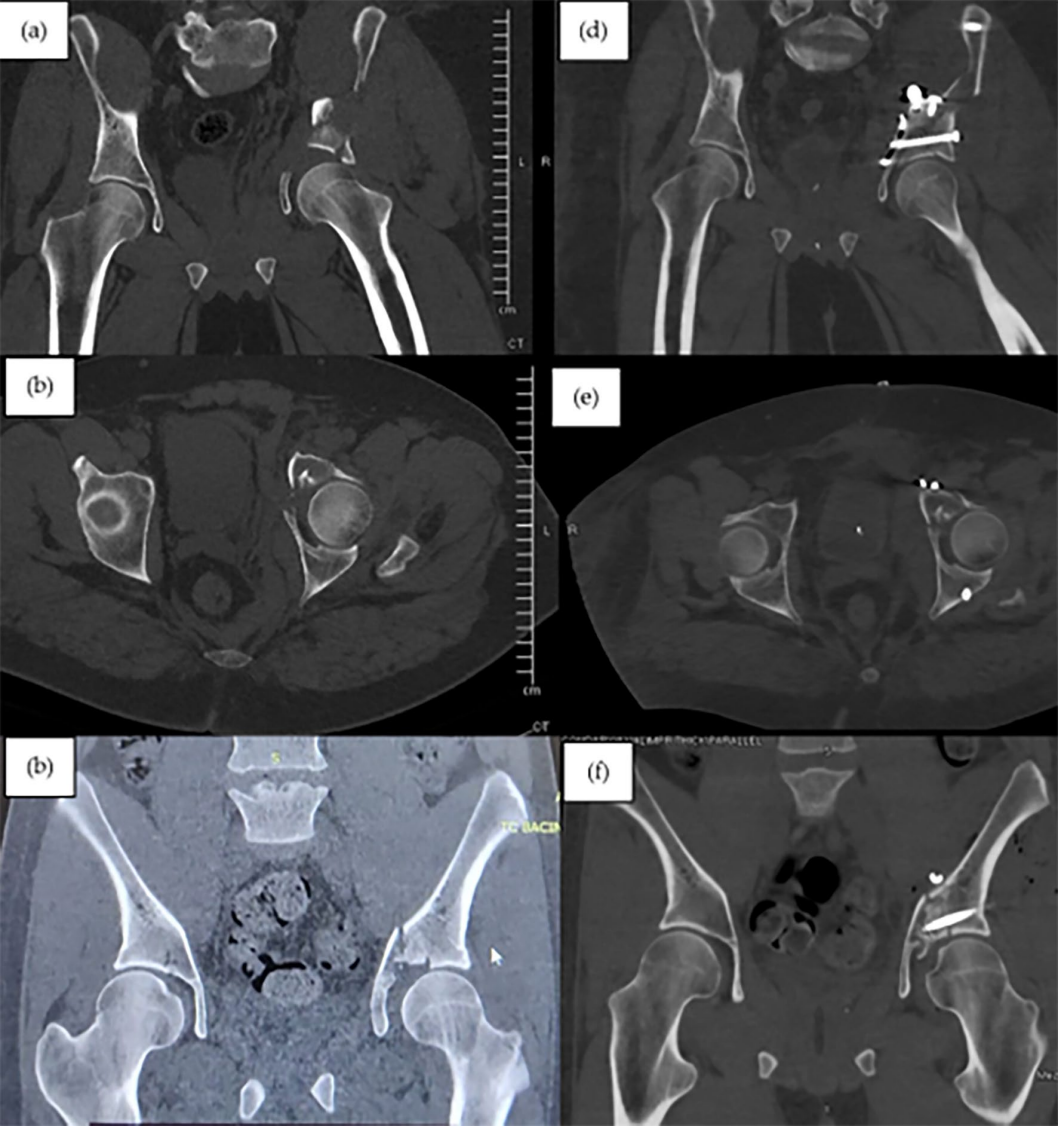
**a**–**c** Preoperative CT scan demonstrating impacted joint fragment of the acetabular roof; **d**–**f** postoperative CT scan after arthroscopic-asssited reduction and fixation, the postoperative protocol was on weight-bearing for 2 months, immediate passive assisted and active progressive range of motion of the hip

Achieving an anatomic reduction of complex acetabular fractures is essential to optimize clinical outcomes, but for certain fracture patterns with impacted articular fragments such a reduction can be challenging [23]. The use of this arthroscopic-assisted technique may assist anatomic reduction as it facilitates direct visualization of the articular surface (Fig. 6).

Matta et al. [4] demonstrated that clinical outcomes are closely related with the quality of the reduction: a poor fracture reduction (> 3 mm) being one of the most negative prognostic indicators followed by multi-fragmentary fractures of the posterior/anterior wall and transverse multi-fragmentary fractures of the tectum. This described technique can ensure an anatomic reduction and the restoration of a congruent acetabular roof.

In the current literature, there are a few case reports describing the use of arthroscopy for acetabular fractures. Kim et al. [24] described an anterior column fracture involved acetabular dome in a 20-year-old patient treated with an arthroscopic-assisted exposure (anterolateral, anterior and posterolateral portals), percutaneous screw fixation, and an open lateral window approach for iliac wing plate fixation. The authors noted a successful reduction with no complications.

Götz and Schulz [25] described a 53-year-old patient with an anterior column fracture and a multifragmentary joint surface that was successfully treated with an arthroscopic-assisted ilioinguinal approach without any complications.

One concern with arthroscopic-assisted fracture reduction in this anatomic area is the risk of fluid extravasation and subsequent compartment syndrome. Shakuo et al. [26] reported abdominal compartment syndrome after an arthroscopic-assisted reduction of an acetabulum anterior column fracture. This patient was treated with percutaneous peritoneal drainage, and they recovered 8 weeks after the operation.

Similarly, Bartlett et al. [20] reported a case of cardiopulmonary arrest after hip arthroscopy was used to remove loose bodies after ORIF of a both-column acetabular fracture, which was contributed to intra-abdominal compartment syndrome caused by fluid extravasation. The patient recovered after cardiopulmonary resuscitation and an emergent laparotomy.

We believe that the risk of fluid extravasation and compartment syndrome is mitigated by the simultaneous AIP approach which allows for drainage of extravasated fluid and have not experienced this complication in our practice.

Surgeons must also be aware of complications specific to hip arthroscopy, including risk of muscle, nerve and vessels injuries, distraction-type injury, acetabular labrum and articular cartilage damage, trochanteric bursitis and iliopsoas tendinitis, as well as the additional risks associated arthroscopy in the setting of fracture, which could involve fluid extravasation, hypothermia, infections, deep vein thrombosis, blood loss, pneumonia, sepsis and multiple organ failure [6].

Based on our experience, arthroscopic-assisted fracture reduction of acetabular fracture is most useful for fractures with impacted fragments of the weight-bearing dome of the acetabulum and fractures with associated osteochondral loose bodies. This technique allows a full acetabular vision in a minimally invasive way compared to a surgical hip dislocation [27].

We have found that this described technique is not useful in cases of fractures that do not have joint impaction, as extraarticular fracture reduction often suffices in obtaining anatomic reductions. Other limitations of hip arthroscopy include intraarticular cartilage damage second to introduction of instrumentation, longer operation times, and the need for surgeons with hip arthroscopy skills, which must be weighed against the benefits of its use. Due to these limitations, this technique may be best reserved for referral centers with high annual acetabular fractures volumes and an expert treatment team familiar with hip arthroscopy.

In conclusion, this paper describes an arthroscopically assisted reduction technique for acetabular fractures with impacted joint fragments that allows for direct visualization and anatomic reduction, maximizing the chances for optimal clinical outcomes.

## Data Availability

All data have been stored in the dedicated repository of University of Turin.
